# Prevalence and risk factors for latent tuberculosis in polish healthcare workers: the comparison of tuberculin skin test and interferon-gamma release assay (IGRA) performance

**DOI:** 10.1186/s12995-021-00326-y

**Published:** 2021-09-01

**Authors:** Monika Szturmowicz, Beata Broniarek-Samson, Urszula Demkow

**Affiliations:** 1 1st Department of Lung Diseases, National Tuberculosis and Lung Diseases Research Institute, Warsaw, Poland; 2 Department of Laboratory Diagnostics, Institute of Mother and Child, Warsaw, Poland; 3 Department of Laboratory Diagnostics and Clinical Immunology of Developmental Age, Medical University of Warsaw, Warsaw, Poland

**Keywords:** Tuberculosis, LTBI, Interferon gamma release assay, Tuberculin skin test, Risk factors

## Abstract

**Background:**

Tuberculosis (TB) is still one of the most common infectious diseases worldwide. Health care workers (HCW) are at particular risk of the disease due to their constant exposure to TB patients or their specimens, nevertheless no specific surveillance is widely recommended in this group of professionals. Both, tuberculin skin test (TST) and interferon-gamma-release-assays (IGRAs) are widely applied to detect latent tuberculosis infection (LTBI). The aim of the present study was to evaluate the prevalence and risks of LTBI in the population of Polish HCW, to identify factors associated with LTBI, as well as to determine the rate of the discordance between the results of the two applied tests in relation to various factors in a TB endemic setting. The study participants were recruited from several health care facilities (hospitals and outpatients clinics) all over the country. Laboratory personnel included 156 persons from both TB and non-TB laboratories (118 clinical pathologists, 38 laboratory technicians), 31 medical doctors, 29 nurses (from both TB and non-T**B** wards and from family practices), 6 other medical employees (patients assistants). Out of examined group 88 (40%) declared constant (everyday) occupational contact with TB patients and/or contagious biologic materials, 134 (60%) reported sporadic (incidental) contact (few times a year). Administrative HCWs who were not in direct contact with patients were not included in the study group.

**Material and methods:**

LTBI status was prospectively evaluated in 222 HCW, 204 females, 18 males, aged 40.8 ± 9 years, with tuberculin skin test (TST) and interferon gamma release assay (QuantiFERON-TB-Gold in Tube – QFT GIT).

**Results:**

TST ≥ 10 mm was found in 58% of HCW, QFT GIT ≥ 0.35 IU/ml in 23%. Nevertheless the relative number of positive QFT GIT in HCW above 45 years of age exceeded those obtained in general population (prevalence of positive QTF test in polish adult population is around 23%). The risk of obtaining positive QFT GIT was significantly increased in the participants older than 44 years (OR = 4.95, 95%CI:2.375–10.193), in those employed > 10 years (OR = 2.726, 95%CI:1.126–6.599), and in those who reported the direct contact with tuberculous patients or infected biological materials (OR = 8.135, 95%CI:1.297–51.016). The concordance between TST and IGRA was poor (kappa 0.23), especially in younger participants, possibly due to BCG vaccination in childhood.

**Conclusion:**

The increased risk of LTBI in Polish HCW was related to age, duration of employment and contact with infectious patients or their biological specimens. TB infection control measures in health care facilities in Poland are still insufficient. It is crucial to increase awareness about the importance of detecting and treating LTBI of HCW.

## Introduction

Tuberculosis is a global disease and the leading infectious cause of death worldwide. One fourth of the world population is infected with *M. tuberculosis* [1]. In about 90–95% of infected persons, tubercle bacilli persist in the organism but do not proliferate (or proliferate only occasionally) and do not cause the development of active tuberculosis [2]. Such a clinical condition is called latent tuberculosis infection (LTBI) [3]. Nevertheless, according to epidemiological estimations in 2018 year, 10 million of people were diagnosed with active tuberculosis worldwide, and 1.2 million – died of this disease [4, 5].

The epidemiological situation of tuberculosis is different in various regions of the world. In 2018 the highest estimated tuberculosis incidence rates were noted in South Asia (220 per 100,000 inhabitants) and in Africa (180 per 100,000 inhabitants) [4]. The lowest incidence was found in the Americas and in Europe, 29 per 100,000 and 28 per 100,000, respectively [4, 5]. The countries with tuberculosis incidence rate lower than 10 per 100,000 are approaching the elimination phase of the disease. In 2017 there were 26 of such countries in the European Union and European Economic Area [5]. This goal is, however, difficult to obtain due to many factors that may cause the reactivation of LTBI [6]. The epidemiological situation of TB in Poland significantly improved over the last 70 years. The notification rate dropped rapidly from 290.4/100000 in 1957 to 17.4/100000 in 2014 [7]. According to the ECDC criteria, Poland is classified as the low-incidence country [7]. The high percentage of Polish TB patients include unemployed and homeless people, alcoholics and drug abusers but not immigrants. Apart from social problems, the delay to diagnosis and treatment of TB contributes to unsuccessful outcomes i.e. the low proportion of patients with positive treatment results. Rowinska-Zakrzewska et al. considered that advanced disease, with more than three lung fields involved, was recognised in around 80% of newly admitted TB patients. The poor outcomes of TB therapy can be explained by decreasing competences of health care workers as well as non-adherence to treatment attributed to poor social conditions, low education level, unemployment, alcoholism, and homelessness [7]. The risk factors of LTBI reactivation include: advanced age, poor conditions of life (malnutrition), addictions (alcohol, narcotics, cigarettes), silicosis, other diseases (HIV, diabetes, renal insufficiency, neoplasms), immunosuppressive therapy (especially anti TNF medications) and corticosteroids [7, 8]. Thus it is very important to recognise and treat LTBI, to prevent active tuberculosis development, especially in patients belonging to the above mentioned risk groups.

LTBI can be recognized by tests based on the ability of sensitized memory lymphocytes and other cells to recognize the antigens of *M. tuberculosis.* Those are tuberculin skin test (TST) and interferon gamma releasing assays (IGRAs) [9]. TST is based on measuring the induration caused by subcutaneous reaction of immunocompetent cells with the antigens included in tuberculin. IGRAs measures the amount of interferon gamma (IFN gamma) released after stimulation of lymphocytes with tuberculous antigens ESAT-6 and CFP-10 (QuantiFERON -TB - Gold in Tube) or by counting, after such exposition, stimulated lymphocytes producing IFN gamma, (T-Spot.TB) [9–12].

.Tuberculin used in TST include crude antigens of *M. tuberculosis* but also antigens of Bacillus Calmette Guerin (BCG). Thus false positive reactions may be observed in persons vaccinated against tuberculosis [11]. IGRAs are more specific, because they are based on measuring the amount of IFN gamma released due to recognition of antigens specific of *M. tuberculosis* and not of BCG [9].

One of the groups with increased probability of LTBI due to occupational exposure to patients infected with *M. tuberculosis* or their specimens are the HCW: doctors, residents, medical students, nurses, social workers and laboratory staff [13]. Generally infection control measures to protect HCW, including personal protective equipment and proper ventilation, undertaken in high or medium incidence countries, are not adequate, and are not a priority [2]. Moreover, HCW were not listed as a separate risk group of infection in the recently published guidance for programmatic management of LTBI in the European Union/European Economic Area [14]. In Poland and in many TB endemic settings, screening for LTBI among HCWs is very limited in practice and preventive treatment is almost never considered. Therefore we aim to increase the awareness of these problems and to provide a clearer understanding of various personal and occupational factors associated with LTBI occurrence in HCWs.

The aim of our study was to evaluate the prevalence of LTBI, assessed by TST and IGRA (QuantiFERON- TB- Gold in Tube) in the population of Polish healthcare professionals, taking into account various sociodemographic and occupational factors, including their exposure to tubercle bacilli, and to compare the LTBI prevalence in the study group with the data obtained in general Polish population assessed within the same period of time. Furthermore, we aimed to determine factors accounting for the discordance between TST and IGRAs in HCWs, in BCG vaccinated population.

## Material and methods

LTBI status has been prospectively evaluated in 222 healthy medical care professionals (every participant was examined by specialist in internal medicine/lung diseases, medical history was taken and physical examination was performed, if needed), 204 women and 18 men, mean age 40.8 +/− 9 years, range 23–69 years. The study participants were randomly selected across several health care institutions (hospitals and outpatients clinics, both TB and non-TB) around the country. In Poland every patient with diagnosed TB is immediately transported to TB hospital. The two methods of subjects recruitment were applied. First, clinical workers involved or not in TB care (doctors, nurses and laboratory workers) from the whole country, undergoing training or participating in the course at National Institute of Tuberculosis and Lung Diseases, were invited to participate in the study. The topic of the training was related to various aspects related to lung diseases, microbiology or laboratory diagnostics. The second group of participants was recruited from National Institute of Tuberculosis and Lung Diseases and from randomly selected health care institution in Mazovia region. In this case the research group (doctor and nurse) visited the selected institution where they collected blood for QTF, performed TST, taken medical history from participant and filled in the questionnaire. The response rate (the consent for the participation) was around 50% among all HCW who were invited to participate. Interestingly, women were more often willing to participate then men. Furthermore, the managers of several preselected TB institutions refused their employees to participate in the study, probably due to potential claims from the personnel and the high costs of specific actions to reduce the risk of tuberculosis transmission in the hospital. All enrolled subjects were asymptomatic. A structured questionnaire was used for risk assessment of LTBI among HCWs, including sociodemographic factors (age, sex, current or past history of other diseases and medications) and factors related to the employment in health care (duration of professional work, occupational position, exposure to TB patients or their specimens), BCG vaccination history, BCG scar measured by professional staff. Nobody from the enrolled subjects had family history of TB.

The study group included: 156 persons working as the laboratory staff (118 clinical pathologists, 38 laboratory technicians), 31 medical doctors, 29 nurses, 6 other medical employees (patients assistants). Administrative HCWs who were not in direct contact with patients were not included in the study group. The selection process to the study group is explained above. Out of examined group 88 (40%) declared constant occupational contact with tuberculous patients and/or contagious biologic materials, 134 (60%) reported sporadic (incidental) contact i. e. few times per year a contact with symptomatic patient before the diagnosis of TB was made or with clinical material from such patients. The participants were assigned to three age categories (23–44, 45–59, and over 60) and to two different subgroups according to their length of employment (below or above 10 years of employment in health care setting).

The study participants have been vaccinated against tuberculosis in the neonatal period and revaccinated after the sixth year of life (according to vaccination schedule in Poland in force until 2005). The characteristics of the study participants is presented in Table 1.

**Table 1:** Demographic characteristics of the study participants

Sex	Numer and percentage of participants	Age group and mean age	Median age
Women	204 (91.9%)	(23–69) 41.0	40.5
Men	18 (8.1%)	(24–55) 38.0	37.0
Total	222 (100%)	(23–69) 40.8	39.5
	**Mean age +/−SD**	**Minimal age**	**Maximal age**
Women	41.0 +/− 9.0	23	69
Men	38.0 +/− 8.9	24	55
Total	40.8 +/− 9.0	23	69
**Length of employment**	**1–10 years**	**11–45 years**	**Total**
Numer of participants	63 (28%)	159 (72%)	222
**Contact with TB in age groups**	**Sporadic**	**Continuous**	**Total**
All HCW	134 (60%)	88 (40%)	222
(0,24]	1 (0.01%)	1 (0.01%)	2
(24,44]	99 (45%)	42 (19%)	141
(44,59]	34 (15%)	40 (18%)	74
(59,70)	0 (0.00%)	5 (2%)	5

The written consent for participation in the study was signed by all individuals. The study was approved by the Bioethics Committee of the Institute of Tuberculosis and Lung Diseases in Warsaw.

### Diagnostic procedure of LTBI testing

#### Interferon gamma release assay

The tests were performed in 222 participants with commercially available test QuantiFERON®-TB-Gold In Tube, Cellestis (QFT GIT). Venous blood was collected into two heparinized tubes: one - coated with ESAT-6, CFP-10 and TB7.7 antigens, and the other containing pure physiological saline with phosphate buffer (negative control). All test procedures were performed according to manufacturer’s instructions. The tubes were flipped upside down several times and incubated at 37 °C for 16–24 h in an upright position. Next, the blood was centrifuged for 15 min at + 4 °C and at a speed of 3000 RPM. Samples were stored at − 40 °C until interferon gamma concentration was determined by ELISA. The assay was performed by a qualified laboratory worker. A positive test result was defined as an IFN concentration of 0.35 IU/ml or more, according to the manufacturer’s recommendations [9]. An indeterminate result was given if the IFN concentration in the negative control was 8.0 IU/ml or more.

#### Tuberculin skin tests (TST)

TST was performed in 222 subjects by intradermal injection of 0.1 ml (2 units) of tuberculin PPD RT23 (Statens Serum Institute – Copenhagen, Denmark) into the dorsal surface of the forearm. The result was read in 220 participants (the 2 participants did not appear for the second visit) after 72 h by measuring the diameter of induration, transversely to long axis of the forearm using a standardized ruler. TST was considered positive in case of diameter of induration of at least 10 mm.

To our knowledge the participants enrolled to the study were not retested with QTF nor TST. We are not aware about development of active TB in any of the participant. Nobody from those who tested positive received TB-preventative treatment as it is not in accord with Polish guidelines for LTBI treatment as described below.

### Statistical analysis

All the calculations were performed using SPSS statistical packages for Windows 12.0 (SPSS, Inc., Chicago, Illinois). The data have been presented as absolute numbers and percentages of subjects with positive test result. The frequency of positive test results in different groups were compared with chi square test or Fisher’s exact test (in case of cell counts being too low in the contingency tables, preventing the use of the chi square test). Logistic regression analysis has been applied for calculating the risk of positive test combined with specific group characteristics. The results are presented as odds ratio (OR), with 95% confidence interval (CI) and *p* value. The agreement between TST values and the results of IGRA values, was analysed using concordance coefficient kappa with 95% CI, and factors associated with discordance. Values of kappa below 0 indicate lack of conformity, 0 to 0.2 - very poor conformity, 0.21 to 0.4 - poor conformity, 0.41 to 0.6 moderate conformity and > 0.61 – significant agreement. Spearman rank correlation was used to test the association between TST and QTF values. The receiver operating characteristic (ROC) analysis was applied to predict the TST and QTF results according to the length of employment in health care and to determine the most appropriate cut-off value. The accuracy of the prediction was measured as an *area under* the *ROC curve* (AUC). If the examined parameter (length of employment) can distinguish between subjects with positive or negative test result, the AUC should be close to 1. AUC close to 0.5 corresponds to a model with no discrimination ability. Statistical significance was defined as *p* ≤ 0.05 for all analyses.

## Results

### TST results

TST induration ≥10 mm was found in 58% of study participants, and equal or larger than 15 mm in 33% of the study group. The relation between TST results and the age of participants is presented in Table 2.

**Table 2:** TST results of 220 HCW in relation to the age category

Age group (years)	TST ≥ 10 mm No (%)	TST ≥ 15 mm No (%)
23–44	73 (52)	35 (25)
45–59	49 (66)	32 (43)
60–70	5 (100)	5 (100)
Total	127 (58)	72 (33)
p (Fisher exact test) in relation to the whole tested group	0.026	0.0001

Significant differences in the frequency of patients with TST ≥ 10 mm, have been found between three different age groups, nevertheless the percentage of positive results exceeded 50% in every age group. If TST ≥ 15 mm was regarded as cut off, more pronounced differences between the age groups were demonstrated, with significantly higher rate of positive TST results in older group, comparing to younger ones. All 5 participants older than 60, had TST result exceeding 15 mm.

The results of TST in relation to the intensity and duration of contact with tuberculosis patients or patients’ specimens are shown in Table 3.

**Table 3:** TST results in relation to the intensity and duration of contact, in 220 healthcare workers

Intensity and duration of contact	TST ≥ 10 mm No (%)	TST ≥ 15 mm No (%)
Sporadic contact	66 (50)	30 (23)
Continuous contact	61 (70)	42 (48)
P (Fisher exact test) in relation to the whole tested group	0.003	0.0001
1–10 years of employment	33 (54)	15 (25)
11–45 years of employment	94 (59)	57 (36)
p (Fisher exact test) in relation to the whole tested group	0.81	0.15

The number of the analysed results is 220 as the 2 subject did not appear for the read of TST result

Positive TST results have been observed significantly more frequently in persons declaring continuous contact with tuberculosis, comparing to those who reported sporadic contact. No relation was observed between TST positivity and length of employment as a healthcare worker.

Logistic regression analysis was used to calculate the risk (associated with increased odds) of positive test result combined with specific group characteristics. If the risk of LTBI, was defined as TST ≥ 10 mm, none of the examined factors reached statistical significance, neither in the whole group of HCWs, nor in selected subgroups of medical doctors, nurses and patients’ assistants. (Tables 4, 5 and 6).

**Table 4:** The association between various risk factors and TST ≥ 10 mm. / HCW, whole examined group, TST ≥ 10

		OR	95% CI		p
Length of employment	(0–10)	1.00			
	(11–45)	1.04	0.56	1.93	0.91
Contact	sporadic	1.00			
	constant	1.96	0.68	5.67	0.21
Exposure to TB	No	1.00			
	Incidental	1.25	0.58	2.69	0.57
	Contagious specimen	1.50	0.46	4.85	0.50
	TB patients	1.29	0.45	3.70	0.63
Sex	F	1.00			
	M	0.61	0.22	1.72	0.35

**Table 5:** Laboratory personnel, TST ≥ 10

		OR	95% CI.		p
Sex	F	1.00			
	M	0.75	0.18	3.02	0.68
Length of employment	(0–10)	1.00			
	(11–45)	1.19	0.58	2.41	0.64
Contact	sporadic	1.00			
	constant	***2.36***	1.21	4.60	***0.01***

**Table 6:** Medical doctors, nurses and patients’ assistants, TST ≥ 10

		OR	95% CI		p
Sex	F	1.00			
	M	0.55	0.10	3.06	0.49
Length of employment	(0–10)	1.00			
	(11–45)	0.70	0.18	2.80	0.61
Occupational position	Doctor	1.00			
	Nurse/patient assistant	1.17	0.31	4.41	0.82
Contact	sporadic	1.00			
	constant	2.77	0.68	11.22	0.15

The risk of TST ≥ 10 was significantly increased (OR = 2.36, 95%CI:1.21–4.60, *p* = 0.012) in the selected group of laboratory workers declaring the constant contact with specimens from TB patients. (Table 4B).

Considering probability of having TST ≥ 15 mm, both clinical staff and laboratory personnel with constant contact with TB patients had elevated risk (odds) for a TST ≥ 15 mm (OR = 6.34, 95%CI:1.38–29.13, *p* = 0.018 and OR = 3.05, 95%CI:1.52–6.11, *p* = 0.002, respectively). The results of logistic regression analysis for TST ≥ 15 mm are presented in Tables 7, 8 and 9.

**Table 7:** The association between various risk factors and TST ≥ 15 mm. HCW, whole examined group, TST ≥ 15

		OR	95% C.I.		p
Length of employment	(0–10)	1.00			
	(11–45)	1.40	0.69	2.83	0.35
Contact	sporadic	1.00			
	constant	1.98	0.66	5.91	0.22
Exposure	No	1.00			
	Incidental	1.05	0.40	2.76	0.93
	Contagious material	1.86	0.51	6.81	0.35
	TB patients	2.96	0.93	9.40	0.07
Sex	F	1.00			
	M	0.59	0.18	1.96	0.39

**Table 8:** Laboratory personnel, TST ≥ 15

		OR	95% CI		p
Sex	F	1.00			
	M	0.99	0.22	4.47	0.999
Length of the employment	(0–10)	1.00			
	(11–45)	1.87	0.82	4.27	0.14
Contact	sporadic	1.00			
	constant	***3.05***	1.52	6.11	***0.002***

**Table 9:** Medical doctors, nurses and patients’ assistants, TST ≥ 15

		OR	95% C.I.		p
Sex	F	1.00			
	M	0.16	0.02	1.34	0.09
Length of the employment	(0–10)	1.00			
	(11–45)	0.69	0.15	3.14	0.64
Occupational position	Doctor	1.00			
	Nurse/patients’ assistant	0.57	0.13	2.38	0.44
Contact	sporadic	1.00			
	constant	***6.34***	1.38	29.13	***0.018***

The above statistical analysis showed that the length of employment did not affect the TST results. Taking into account the assumption that the selected cut off value (10 years) may not be appropriate, we have performed receiver operating characteristic (ROC) analysis to predict the TST result according to the length of employment in health care. The optimum cut-off point derived from the applied analytical method (finding the cut-off point on a ROC curve) cannot be found (data not shown). Furthermore the area under the ROC curve showed that the length of employment cannot be used as a criterion to measure the TST discriminative ability for both TST ≥ 10 mm and TST ≥ 15 mm.

The area under the curve ranges from 0.588 to 0.595, corresponding to a model with no discrimination ability. The analysis does not support the use of the parameter „length of employment” as a predictor of TST result.

### QuantiFERON -TB –gold in tube results

Positive results of QTF GIT (≥0.35 IU/ml) had been obtained in 27% of participants. The frequency of positive QFT GIT results was significantly associated with the age of HCWs. Similarly to TST the highest percentage of positive test results was noted in the oldest group of patients i.e. above 60 years (80%). The differences between tested groups were statistically significant (*p* = 0.0001). The relation between QFT GIT results and participants age, is shown in Table 10.

**Table 10:** QFT GIT results in relation to the age in 222 healthcare workers. Positive and negative test results are presented as an absolute number and percentage of cases in different age categories

Age group (years)	QFT ≥ 0.35 No (%)
23–44	20 (14)
45–59	36 (49)
60–70	4 (80)
Total	60 (27)
p (Fisher exact test) in relation to the whole tested group	0.0001

QFT GIT results in relation to the intensity and duration of the contact with tuberculosis is presented in Table 11.

**Table 11:** QFT GIT results in relation to intensity and duration of contact with *M. tuberculosis* in 222 healthcare workers

Intensity and duration of contact	QFT ≥0.35 IU/ml No (%)
Sporadic contact	19 (14)
Constant contact	41 (47)
p (Fisher exact test) in relation to the whole tested group	0.0001
1–10 years of employment	8 (13)
11–45 years of employment	52 (33)
p (Fisher exact test) in relation to the whole tested group	0.004

Positive result of QFT GIT was found significantly more frequently in the participants declaring constant contact with tuberculosis compared to subjects with sporadic contact. Accordingly, the highest percentage of positive results (47%) was noted in the group with continuous contact with patients or patients’ specimens. The differences between the groups were significant, *p* = 0.0001.

Furthermore, the frequency of positive test result was significantly higher in those employed for more than 10 years than in those working in health care institutions for less than 10 years. Using logistic regression analysis, the risk of obtaining positive QFT GIT test, combined with specific group characteristics, is presented in Table 12. The results are presented as odds ratio (OR), with 95% confidence interval (CI) and *p* value. The risk of positive QFT GIT was increased fivefold in the participants 45–59 years old, and twelvefold in those 60–70 years old, comparing to those aged 23–44 years. The risk was also increased by 2.7 times in those participants who have been employed in medical institution for more than 10 years, by 8 times in those who declared the contact with TB patients samples, and even by 17 times in those who had constant contact with tuberculous patients.

**Table 12:** The risk of positive QTF GIT test according to different factors in the group with 222 healthcare workers

Factor	OR	95%CI	p
**Age (years): 23–44 vs**			
45–59	4.92	2.37–10.19	0.0001
60–70	12.31	1.26–120.72	0.031
Employment:			
**> 10 years** vs < 10 years	2.73	1.13–6.6	0.03
Contact:			
**Sporadic** vs continuous vs	2.27	0.7–7.36	0.17
Type of contact:			
**Sporadic** vs			
TB patients’ specimens	8.13	1.30–51.02	0.025
Patients with tuberculosis	17.07	3.13–93.02	0.001

The reference category is bolded

The analysis performed in the group of laboratory personnel showed that the risk of positive QFT GIT was increased in the group of subjects working with TB patients specimens, and in those working in health care for more than 10 years (Table 13).

**Table 13:** The risk of positive QFT GIT according to different factors in laboratory personnel

Factor		OR	95% C.I		p
Sex	F	1.00			
	M	0.64	0.11	3.57	0.61
Length of employment	(0–10)	1.00			
	(11–45)	***3.60***	1.37	9.45	***0.009***
Contact	sporadic	1.00			
	constant	***3.91***	1.87	8.17	***0.000***

The employment at TB departments almost 16 times increased the risk of having QTF > 0.35 in the group of doctors and nurses or patients assistants. The length of employment at the TB ward did not increase the probability of having QTF > 0.35 (Table 14).

**Table 14:** Risk of positive QFT GIT according to different factors in the group of doctors, nurses or patients’ assistants

Factor		OR	95% C.I.		p
Sex	F	1.00			
	M	0.00	0.00	–	0.99
Length of employment	(0–10)	1.00			
	(11–45)	1.36	0.16	11.26	0.77
Occupational position	Doctor	1.00			
	Nurse/assistant	0.68	0.112	4.01	0.67
Contact	sporadic	1.00			
	constant	***15.869***	2.729	92.286	***0.002***

Similarly to TST, we have performed receiver operating characteristic (ROC) analysis to predict the QTF result according to the length of employment in health care. The optimum cut-off point derived from the applied analytical method (finding the cut-off point on a ROC curve) cannot be found (data not shown). Furthermore the area under the ROC curve showed that the length of employment cannot be used as a criterion to measure the QTF discriminative ability.

The area under the curve (higher for QTF than for TST) indicated that the parameter „length of employment” had better discriminating ability as a predictor of QTF result than TST result, however the analysis does not support the use of this parameter as an accurate predictor of QTF result.

#### The concordance of the TST and QFT GIT results

In whole tested group of 220 HCW the pairwise concordance rate between QTF GIT and TST > 10 mm was poor κ = 0.23 (95%CI: 0,12 – 0,33). The discordant results were noted in 128 persons (58.2%), concordant in 92 subjects (41.8%).

The agreement between QTF GIT and TST cut off ≥15 mm was moderate - kappa value reached 0.40 (OR 95%Cl: 0.28–0.54). Both tests were negative in 127 persons (57.7%), and both positive in 38 (17.3%) subjects.

**Fig. 1:**
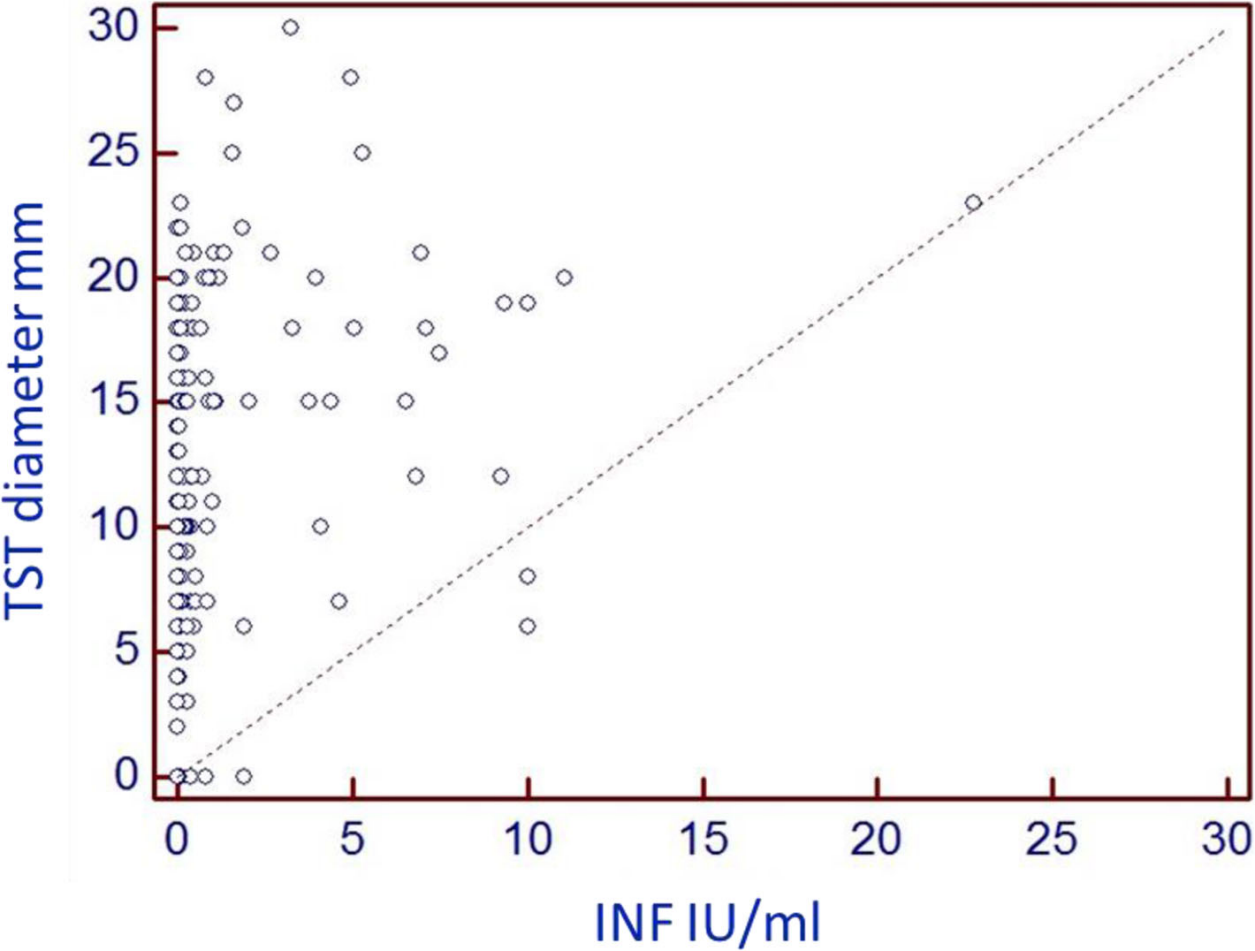
Correlation between TST and QTF GIT results

To investigate the correlation between the TST and the QFT GIT result, we used Spearman’s coefficient of rank correlation. The results are presented at Fig.1.

A moderate positive correlation between both results was obtained for sample size of 220 subjects, as shown by the Spearman’s rank correlation coefficient (0.46, 95% confidence interval 0.35 to 0.56) (*p* < 0.0001).

## Discussion

The risk of nosocomial infection with *M. tuberculosis* due to significant exposure to patients with active tuberculosis and their specimens, together with the insufficient use of protective equipment and inadequate ventilation of working areas, remains an important problem in healthcare settings.

The authors of the present study prospectively assessed the prevalence of LTBI in healthy HCW by means of QTF GIT and TST, in Poland. As far as we know, there was no other multicentre evaluation of LTBI prevalence and its related risk factors among HCWs in Eastern Europe. All participants were BCG vaccinated at least twice in their life: in the neonate period, and above the sixth year of age. Therefore, according to published recommendations, the method of choice to assess LTBI among BCG vaccinated HCW should be an IGRA test [14]. Nevertheless TST results were analysed as well, as some investigators indicate that TST is more sensitive than IGRA, although less specific, in the vaccinated population [15]. According to Polish National Anti-Tuberculosis Program [16], the TST induration ≥10 mm is considered positive, while other studies [17] and guidelines developed by Centres for Disease Control (CDC) and the American Thoracic Society (ATS) assume that for individuals who are not in high risk groups, LTBI can be diagnosed if TST induration is ≥15 mm [18, 19].

In our study positive results of TST (≥10 mm and ≥ 15 mm) were found in 58 and 33% participants of the study respectively, positive QTF GIT results (≥0.35 IU/ml) in 27%. The overall prevalence of LTBI in medical staff was found to be consistent with LTBI prevalence in 700 healthy Polish adults from randomly selected family practice, investigated in the same period of time [20]. In this study Kuś et al. found that 50% participants had TST ≥ 10 mm, 26% had TST ≥ 15 mm and QFT GIT ≥0.35 IU/ml was detected in 23% of enrolled subjects [20]. The comparison of these results revealed that the overall prevalence of LTBI in Polish HCW was only slightly higher than in general Polish population.

The analysis of TST results in selected age groups, performed by Kuś et al., revealed that the test was more often positive in younger participants comparing to older ones: 55% of those in the age group of 25–44 years, 52% of those aged 45–59 years, and 35% of those above 60 years of age [20]. On the contrary, positive QTF GIT results were found more often in the older participants, comparing to younger ones: 14% of those 25–44 years of age, 33% of those aged 45–59 years and 49% of those above 60 years of age [20]. Results of LTBI assessment with QFT GIT, were in agreement with epidemiological data of tuberculosis in Poland. In the same period, tuberculosis incidence rate in general population was 19.7 per 100,000, significantly lower incidence rate was found in young comparing to older age groups: 14.3 per 100,000 among those 22–44 years of age, 35 per 100,000 in those aged 45–64 years, and 38 per 100,000 in those above 65 years of age [21].

In the presently investigated group of Polish HCW, opposite to the general population of Polish citizens, the percentage of participants with TST ≥10 mm and TST ≥ 15 mm have been increasing with age, moreover the differences between age groups were much more pronounced if TST ≥ 15 mm was regarded as a cut-off. This original observation suggests that the impact of occupational contact with *M. tuberculosis* in HCW prevails the effect of prior BCG vaccination on TST results.

The percentage of positive QFT GIT results in the groups of medical staff aged 45–59 and 60–70 years, were very similar to those with TST ≥ 15 mm. Large discrepancy was found between TST ≥ 15 mm and QFT GIT in healthcare workers aged 23–44 years. Based on IGRA test, LTBI would be diagnosed in 14%, based on TST ≥ 15 mm – in 25% of them. We assume, that this difference was mainly due to vanishing reaction to past BCG vaccinations in older HCW, comparing to younger ones. This is consistent with the guidelines of ECDC, stating that TST reactivity caused by BCG usually wanes with time [22]. We can also conclude that TST ≥ 15 can be used as LTBI marker comparable with IGRA in individuals over 45 years, vaccinated with BCG in childhood. QTF GIT is preferred for LTBI diagnosis in adults younger than 44 years as the results of TST in this group are confounded by BCG vaccination [23].

It is worth to notice, that the prevalence of positive IGRA results in medical caregivers was 49% in those aged 45–59 years and even 80% - in those aged 60–70 years, compared to 33 and 49%, respectively - in general Polish population [20]. Therefore we conclude that despite the similar overall prevalence of LTBI assessed by QFT GIT in both populations, the prevalence of LTBI in medical staff aged 45 years or more, was higher than in age-adjusted general population.

An effort was made to compare the LTBI prevalence measured with QFT GIT in Polish HCW with the results obtained with the same method and in comparable period of time, in medical staff of other countries [24–30].

The results of those studies, indicate that LTBI prevalence in the population of HCW is influenced by epidemiological situation in the country, but also, to the great extent, by the participants’ age, place of birth, the work place, and family history. Thus, these populations shouldn’t be directly compared.

In our study, LTBI risk, as evidenced by QFT GIT results, in the entire group of HCW was age-dependen but also differed with respect to factors such as the length of employment, as well as the intensity and duration of the exposure to TB patients or their specimens. The risk of obtaining positive IGRA in medical workers was increased by 3 times - in those above 44 years of age, by 5 times - in those employed for more than 10 years, and by 8–12 times in those declaring continuous contact with TB patients and their specimens. Recently published data from South Korea revealed that LTBI risk in medical staff of tertiary hospital was independently connected with age of participant, but not with length of occupation [31].

Logistic regression analysis revealed that the odds ratio, i.e. the possibility of having TST induration of ≥10 mm and TST ≥15 did not differ between all selected subgroups of the total population of enrolled subjects. Only in the group of laboratory personnel we confirmed 2.36 times higher risk of having TST ≥ 10 mm and 3 times of TST ≥15 associated with continuous contact with clinical specimen. In the group of HCW carrying for TB patients, the risk of having TST ≥ 15 was 6.338 times higher than in the group of workers from non-TB wards. This observation is consistent with the study by Kuś et al. who claimed that TST is not a reliable test for LTBI detecting in Polish population [20]. Major reason for this could be lower specificity and false positivity associated with TST in BCG vaccinated population, or with occupational or environmental exposure to non-tuberculous mycobacteria (NTM) influencing TST results. Tuberculin is a mixture of antigens common to a variety of mycobacterial species. The hydrophobic NTM can be readily aerosolized causing NTB sensitisation in health-care settings [32]. These results, in comparison to previously discussed TST results, support the usefulness of the QFT GIT as an universal tool for the detection of LTBI in Poland [33].

In our study the cut-off point for continuous variable “the length of employment” (10 years) was arbitrarily selected. Therefore we considered necessary to determine if an optimal cut-off point to stratify enrolled subjects with higher degree of objectivity, can be found. According to ROC analysis the optimal cut-off point for the parameter “length of employment” to predict the TST result (≥10 and ≥ 15) or QTF GIT (≥0.35 IU/ml) could not be found. The „length of employment” has better discriminating ability as a predictor of QTF result than TST result (as measured by the area under the curve), however the analysis does not support the use of this parameter as an accurate predictor of QTF GIT result.

Interestingly, according to the logistic regression analysis the risk of being latently infected with TB was significantly higher in clinical staff (OR = 17.071) than in laboratory personnel (OR = 8.135). This may point to the better protective measures in TB laboratories in comparison to tuberculosis wards in Poland. On the basis of our results we conclude that there is an urgent need to implement effective procedures and precautions to reduce the risk for exposure to airborne pathogens for managing patients who may have active TB.

Our results showed that TB transmission in healthcare facilities in Poland is high. We may speculate that it can be due to ineffective control measures in dedicated care facilities. The precautions for laboratory safety, such as local exhaust ventilation and laboratory hoods, are more effective but still insufficient in Polish TB laboratories. On the other hand, the analysis of our results showed that the TB transmission in healthcare facilities is steadily decreasing as LTBI prevalence is significantly lower in those employed for less than 10 years as HCW. The modernisation of old buildings from XIX century, hosting most of TB departments in Poland, enables implementation of more effective interventions to improve ventilation in existing infrastructure.

It is also important to notice, that despite great reduction in tuberculosis incidence rate, prevalence of LTBI in HCW has not been reduced proportionally, suggesting the need of further surveillance in this group, especially in those working in high-risk settings and the implementation of evidence-based infection control practices. Therefore LTBI screening should be strongly advised in Polish medical staff, especially in those exceeding 44 years of age, or those employed for a long time with the direct contact with infective materials or patients. QTF can be recommended for serial testing as boosting effect does not occur, contrary to repetitive tuberculin administration.

Both TST and IGRA are based on immune response of activated antigen presenting cells and sensitized lymphocytes to *M.tuberculosis* antigens. The TST rely on the reaction to intradermal injection of tuberculin, which induces a local inflammatory induration that can be measured, whereas IGRAs are in vitro tests measuring IFN-γ released from CD4+ lymphocytes upon stimulation by ESAT-6 and CFP-10, two antigens encoded in the region of difference 1 locus present in *M. tuberculosis* genome and also present in tuberculin. Therefore, additionally to the comparison of tests characteristics, we aimed to directly correlate the surrogate markers of the adaptive immune response to tuberculin antigens as measured by TST induration or IFN-γ production. A moderate positive correlation between both results was obtained confirming that, even though both test do not measure the same components of the immunologic reaction, both are a part of the same a complex and multifaceted immune response developed during *M. Tuberculosis* dormancy. As mentioned before, the data about incidence of LTBI in polish HCW is scarce, the available analyses are limited in scope and the collected data come from single institutions in Warsaw [34, 35]. Our findings advocate for widespread screening of HCW exposed to active TB patients or their specimens. Furthermore, the risks and benefits of LTBI therapy at the individual level need to be carefully balanced, considering an increased risk of progression to overt disease versus drug-related side effects. Polish recommendations for preventive treatment for LTBI apply only to persons with LTBI and: HIV-infected, those who are on immunosuppressive therapy (i.e. TNF-α antagonists). organ transplant recipients, children and adolescents. The isoniazid in dose 5 mg/kg is administered for 6 months to these patients [21]. The LTBI treatment to everyone with a positive QFT or TST is not recommended in Poland. Our results strongly suggest that employers should be encouraged to implement and monitor occupational infection control strategies for preventing TB transmission, such as specific procedures, environmental controls in healthcare facilities and use of personal protection equipment [23]. The prevalence of LTBI and active TB in HCWs compared with the general population should be monitored, to ensure that sufficient protective measures are in place.

According to ECDC report, weak evidence showed no increased risk of LTBI, but an increased risk of active TB in all HCW compared to the general population. Analysis showed that LTBI screening of all HCW is not likely to be cost-effective, except for individuals working with active TB patients or their specimens [22]. These statements are consistent with our findings.

## Conclusions

1. LTBI among HCWs in Poland, exceed the background level of the general population.
2. There is limited value in performing TST to detect LTBI in Polish HCW. IGRA is a superior tool over TST, in those regions where BCG vaccination is mandatory.
3. Both occupational and non-occupational factors are associated with LTBI in HCW. The contact with active TB patients has been identified as major risk factor for LTBI in Polish HCW.
4. TB infection control measures in health care facilities in Poland are still insufficient. It is crucial to increase awareness about the importance of detecting and treating LTBI of HCW

## Data Availability

The authors agree that any materials, data and associated protocols promptly that are requested by others are available to readers at request from corresponding author without undue qualifications.
